# Emotional drive in sexual behavior: an exploratory assessment of anchored hedonic values and profiles

**DOI:** 10.3389/fpsyg.2026.1578265

**Published:** 2026-02-19

**Authors:** Carlos Velo Higueras, Sara Domínguez-Rodríguez, Miguel Ángel Ruiz Díaz

**Affiliations:** 1 Universidad Europea de Valencia, Faculty of Health Sciences, Psychology Department, Valencia, Spain; 2 Universidad Europea de Madrid, Faculty of Biomedical and Health Sciences, Biosciences Department, Madrid, Spain; 3 Universidad Autónoma de Madrid, Faculty of Psychology, Social and Methodology Department, Madrid, Spain

**Keywords:** emotional arousal, emotional drive, individual differences, personality, sexual behavior, sexual intercourse, subjective arousal

## Abstract

**Introduction:**

Prior literature has shown how emotional variables applied to sexual behavior can be differentiated into two dimensions: an emotional context (EC) as a processing state and emotional drive (ED) as a motivation specifically oriented to each stimulus. Following this rationale, we aim to explore the full picture of ED values and profiles in an anchored repertoire of sexual behavior.

**Methods:**

A survey was conducted using an *ad hoc* questionnaire systematically developed based on a prior review. Approaching and avoiding ED and dissonance scores were defined and summarized. An unsupervised variable selection was performed to detect the most discriminative factors according to the Bayesian Information Criterion (BIC). Cluster analysis was performed on ordinal data using Latent Block Model co-clustering.

**Results:**

The average scores showed a sample difference in responses, either in approaching (*t* = −9.86, *p* < 0.01) or avoiding values (*t* = −9.56, *p* < 0.01), finding that a higher ED was the most common in both factors. This difference was also found in reporting other behaviors with a higher than low dissonance (*t* = −11.28, *p* < 0.01), although its absence was the most common outcome. The variable selection revealed that, unlike sets of behavior, accounting for more than 99% of the variance. The cluster analysis yielded four clusters and eight classes for approaching ED and three clusters and six classes for avoiding ED.

**Discussion:**

Our results reveal the importance of considering both positive and negative ED within an exhaustive repertoire and the usual experience of dissonance in the motivation of sexual behavior. All these variables must be considered separately to obtain a full picture and create proper profile descriptions.

## Introduction

As deeply described in one of the most comprehensive anthologies of behavior explanation and analysis, written by Sapolsky (2017), emotion is, in a wide range, an unavoidable part of human information processing and behavior. However, this relevance in behavioral explanations does not mean that it constitutes a clear and developed factor in every research topic. In contrast, despite more than 90 definitions being proposed in the twentieth century, a consensus among scholars and theoreticians has been repeatedly questioned (Plutchik, 2001).

Although the need to introduce emotional factors in the models defining human acts in any field of study is widely acknowledged, some problems arise with labeling and setting boundaries among emotions and accurate substrate involvement (Sapolsky, 2017; Plutchik, 2001; Susanto et al., 2020). Consequently, to overcome these limitations, the rationale for how emotions are introduced and emotional variables are defined is of remarkable importance in model development.

In the field of sexual behavior, in terms of emotion coding, the need to discriminate between two specific subfactors has been previously described. This distinction implies a difference between sexual excitation-inhibition/excitation and affective responses (Moura et al., 2020). In other words, from another proposal, they are called “sexual arousal” and “emotional response” (Nobre and Pinto-Gouveia, 2008), respectively. In fact, in the current literature, this classification has been reported under different terms but has a commonly shared definition:

First, emotion may refer to the processing state or general mood of people when they are thinking, expecting, or having sex. In Cromwell et al.’s (2020) words, it is “the organized affective and behavior-promoting reactions to significant internal and external events” (p. 205), hereafter referred to as the emotional context (EC).

In many possible situations in life, the emotional context and its dynamic changes impact how we perceive, experience, and learn (Sapolsky, 2017). Sexual intercourse has been described as having the power to modulate drive, pleasure, and appraisal (Basson, 2003; Hiller, 2004; Nobre and Pinto-Gouveia, 2008). Citing Hiller (2004, p. 408), “sexual behavior draws together primitive needs for interpersonal contact, security, and reproduction, and elicits powerful emotions from euphoria to destructive urges.”

Second, considering this rationale, if this processing state identified as EC can modulate the level of drive, the latter necessarily conforms to another related but different dimension of drive. Indeed, it is not just speculation but a variable that has been studied, albeit labeled differently for a shared rationale, such as emotional arousal (Madan, 2017), positive/negative affect (Ferrey et al., 2012), or subjective arousal (Kobayashi et al., 2021). In fact, it matches the attitude axis (pleasantness-versus-disgust dimension) from the revisited hourglass model (Susanto et al., 2020), and Cromwell et al. (2020) define motivation as a “process integrating diverse inputs used to form behavioral responses aimed at satisfying needs as well as determining outcome valuation or desired end states of action” (p. 205).

Additionally, some neuropsychological studies have previously reported results of neural networks involved in processes effectively differentiated by a positive or negative load of emotion toward several elements (Cromwell et al., 2020; Hamann et al., 2002; Kim and Hamann, 2007; Sorinas et al., 2019). This drive is defined as the motivational value experienced when a specific stimulus is perceived (Kobayashi et al., 2021). It is important to alter the reward effects (Madan, 2017) and contain accurate information in terms of approaching/avoiding motivation for each input (Blanc et al., 2018).

In the same line of every personality tendency, this emotional drive (ED) differs among individuals. In light of previous results, it has been shown how ED can be expressed differentially by gender, personal objectives, or even by the time-term of their objectives (Kobayashi et al., 2021) and can also lead to attention bias in the processing of peripheral information (Madan, 2017). It has even been reported how a modulation in the response can cause changes in an individual’s ED to stimuli (Driscoll et al., 2018; Ferrey et al., 2012).

There are results on the strong but not exhaustive positive correlation between ED and physiological arousal (Kobayashi et al., 2021) and between ED and the motivation to behave (Driscoll et al., 2018; Ferrey et al., 2012). In short, ED increases the likelihood of activating or acting but does not ensure it. In conclusion, in Cabanac’s words (Cabanac, 2002, p. 80), ED is the “common currency […] any mental experience with high intensity and high hedonic content [pleasure/displeasure].” Therefore, when applied to sexual behavior, this ED would be defined as the hedonic tendency leading to the approach or aversion of a specific element perceived in the sexual scope of the individual.

The logical hypothesis, in light of this evidence, is that each individual may hold a personal drive toward different stimuli in the sexual sphere, thereby forming a personal hedonic tendency. This tendency or pattern includes desires as well as the rejection of sexual behaviors or stimuli.

It is important to note the concept of personal patterns within the whole set of possibilities regarding Yannakakis et al.’s (2021) rationale, which supports measures of emotional intensity, mainly ordinal scales, as good categorizers, especially when anchored to a reference point. In their own words: “[…] experience with stimuli gradually creates our internal context, and discussions of anchors, against which we rank any forthcoming stimulus or perceived experience.”

When exploring a so-called “personal hedonic pattern” of desires and rejections useful to enable comparisons among subjects, it may be especially relevant to use a commonly shared set of words to measure the same elements, where individuals may anchor and offset their responses. To date, we have not found any studies that explore the overall profile of ED in individuals in a sexual behavior context or any that analyze dissonance as a potentially relevant factor in the study of emotional impulses.

Although the aforementioned research does not screen for the emergence of dissonances in the ED, the experience of opposite feelings or thoughts over one sole element has been described in other sexual decision-making (Mannberg, 2012) and appraisals (Demarchi et al., 2020) and should be included in an ED assessment.

Hence, a comprehensive assessment of the current ED applied to specific sexual stimuli must draw a personal profile of hedonic trends in a given set. Considering this substantiation, we conducted the present study under the hypothesis that people experience different subjective ED toward several possible sexual stimuli conforming to a repertoire selection, which may also include the appearance of dissonances.

This study aims to explore this sexual ED profile and screen for any differences within and among subjects in a delimited sample. An additional objective was to describe the distribution of dissonance, if found.

## Methods

### Design

We conducted a single-sample observational cross-sectional study using an online survey. Two independent ethics committees approved the study: the Universidad Autónoma de Madrid committee and Hospital 12 de Octubre in Madrid, Spain.

### Sample and procedure

Although age categorization in sexual behavior is always a cumbersome issue, a recruiting age range of 18–29 was selected, as it has been described as a common factor in adults’ intercultural and sexual development (Wellings et al., 2006) and also in other areas of behavior and physical health (Dowda et al., 2003).

Subjects were selected using incidental sampling from students, patients, and people within the hospital services involved in the project, either from external centers or in different outreach activities organized by the cohort research team, as well as through the snowball method from already recruited individuals, looking for an unbiased composition of the sample. All participants signed an informed consent form and were at least 18-year-old Spanish speakers who could be contacted by email and had not been diagnosed with any impairment that could prevent them from understanding and answering the questionnaire.

All participants were contacted via email. Once the consent form was signed, they were provided with a link to the online questionnaire, and only one attempt was allowed.

### Materials

An *ad hoc* questionnaire was used based on the theoretical proposal according to which any sexual situation described in the literature to date may be classified and placed within a total set (Velo et al., 2023), but it was adapted to enable responses not about experience but ED on every single item, both positive and negative.

It included four areas (mate selection, specific behaviors, other modifying variables, and paraphilias) for different types of stimuli. The rationale and detailed variables can be accessed from the original source (Velo et al., 2023).

For the definition of ED, we avoided the use of emotional colloquial terms because, although they may be commonly used in daily life, they would not necessarily be accurate descriptors. They may be inconsistent and misleading, and could potentially bias the collection of information (Brooks et al., 2016).

Therefore, based on the theoretical framework of approaching-avoiding (Muise et al., 2018) or activation–inhibition (Janssen et al., 2002), we assessed hedonic perception in terms of positive (attraction) and negative (rejection) values and asked the participants to indicate three ordinal scores: none, some, or high. Both considerations were plotted simultaneously to control for the possibility of dissonance.

We scored the dissonance variable for approaching/averting responses. We identified three possible cases: no dissonance (0), when one or two of the two options were at the lowest level; low dissonance (1), when one of the options was scored higher, but the other was also marked over the lowest level; and high dissonance (2), when avoiding and approaching were scored with the same intensity.

Additional questions were included referring to socio-demographic features such as age, biological sex, self-identified gender, academic level, and household income, as well as general indicators of sexual experience, such as overall satisfaction, perceived self-attractiveness, perceived mastery, the number of sexual intercourses per month during the last year, the number of partners in the last 12 months, and having experienced any potentially traumatic event, i.e., any sexual event experienced in an especially intense negative way, with helplessness, frustration, pain, or self-perceived risk.

### Statistical analysis

Demographic features were summarized using the mean and standard deviation for quantitative variables and counts and percentages for categories.

Approaching and avoiding scores for each description of sexual behavior were summarized separately, using both the mean and standard deviation. In addition, dissonance was described as the percentage of low and high levels.

To assess a global picture of the subjects within the whole repertoire, any differences between the individuals’ average scores for the levels of “some” and “high” were assessed using a related sample *t*-test. Approaching and avoiding were analyzed separately again to describe the trends correctly and explore differences. The same analysis was performed using the average dissonance scores.

The general indicators of sexual experience were described using the mean and standard deviation for quantitative variables and the count and percentage for qualitative variables. Association analyses (Rho, Pearson, and Chi-Square) were performed to screen for the association between these general indicators and dissonance levels.

Variable selection was performed to identify the most discriminative items in the set of variables for this population sample. We performed an unsupervised variable selection without parameter estimation using a Gaussian mixture model with conditional independence assumed, implemented in the VarSelLCM R package (Marbac and Sedki, 2017).

Cluster analysis was performed using a set of selected discriminative behaviors. Two separate analyses were performed for attraction and rejection scores. The clusters were obtained using the Latent Block Model co-clustering for ordinal data modeled by the Binary Ordinal Search distribution.

Cluster analysis has been reported as appropriate to assess latent classes or blocks, aiming at goodness-of-fit even over a sample size (Breckenridge, 2000; Marbac and Sedki, 2017). As no clear guidelines have been reported on sample size adequacy in the use of these models (Park and Yu, 2018; Weller et al., 2020), the analysis was carried out, reporting a higher ICL-BIC.

An SEM-Gibbs algorithm with 120 iterations, 80 burn-in iterations, and k-means initialization was used to infer the parameters of the model. The integrated complete data likelihood-Bayesian information criterion (ICL-BIC) was used to select the number of co-clusters or blocks (Marbac and Sedki, 2017). The ordinalClust R package was used for the analysis.

## Results

A total of 345 subjects were recruited from all participating centers. Of them, 207 answered all the characterization questions, and 138 were finally included in the study with a complete set of hedonic values, 27.5% males (Table 1).

**Table 1:** Sample description.

Variable	Distribution
Age (mean, standard deviation)	23.6; 3.32 (18–29 years)
Biologic sex (n, %)	
Male	38 (27.7%)
Female	99 (72.3%)
Gender (self-identified, mostly fitted) (n, %)	
Masculine man	40 (29%)
Feminine man	2 (1.4%)
Masculine woman	5 (3.6%)
Feminine woman	87 (63%)
Trans woman (man born)	1 (0.7%)
Agender (neutral)	3 (2.2%)
Academic level (n, %)	
Basic	2 (1.4%)
Medium professional bachelor’s degree	48 (34.8%)
High professional degree or university degree	43 (31.2%)
University postgraduate	45 (32.6%)
Household income (n, %)	
<20 k per year	61 (44.5%)
20–25 k per year	30 (21.9%)
25–30 k per year	26 (19%)
>30 k per year	20 (14.6%)

Up to 92.1% of biological males identified themselves as masculine males, and 86.9% of biological females perceived themselves as feminine females (Table 1). A total of 34.8, 31.2, and 32.6% had completed a medium, high, or postgraduate level of education, respectively. Overall, 44.5% belonged to homes with an income of less than 20 thousand euros per year, 21.9%, from 20 to 25 thousand, 19% from 25 to 30 thousand, and 14.6% received an income of more than 30 thousand euros per year.

Table 2 shows the distributions of Approach, Reject, and Dissonance by item and tests on the difference between the first and second. Only four items were found to have no significant differences (*p* < 0.05) (Table 2).

**Table 2:** Descriptive results for each variable of interest in the sample.

Behaviors		Approach (M; SD)	Reject (M; SD)	t (sig.)	Dissonance* (%)		
					None	Some	High
Partner-type trait	Biological man trait	2.51 (0.83)	1.38 (0.74)	<0.01	97.2	1.4	1.4
	Biological woman trait	1.99 (0.87)	1.62 (0.80)	<0.01	88.4	0.0	11.6
	Masculine attitude trait	2.39 (0.78)	1.38 (0.65)	<0.01	87.0	2.2	10.9
	Feminine attitude	1.85 (0.87)	1.74 (0.84)	0.41	87.7	0.7	11.6
	Athletic body type	2.73 (0.49)	1.18 (0.45)	<0.01	86.2	2.9	10.9
	Lean body type	2.34 (0.61)	1.29 (0.48)	<0.01	78.3	2.2	19.6
	Plump body type	1.68 (0.67)	1.82 (0.74)	0.20	75.4	0.7	23.9
	Casual partner	1.98 (0.74)	1.76 (0.75)	0.05	68.1	5.1	26.8
	Trusted partner without an emotional bond	2.54 (0.62)	1.31 (0.53)	<0.01	94.2	0.7	5.1
	Steady relationship partner	2.86 (0.42)	1.09 (0.36)	<0.01	77.5	4.3	18.1
	Child age	1.04 (0.25)	2.86 (0.50)	<0.01	99.3	0.0	0.7
	Adolescent age	1.41 (0.69)	2.41 (0.78)	<0.01	84.1	2.9	13.0
	Young adult age	2.79 (0.52)	1.21 (0.53)	<0.01	88.4	0.7	10.9
	Mature age	2.13 (0.73)	1.72 (0.72)	<0.01	60.9	10.1	29.0
	Elder age	1.04 (0.26)	2.85 (0.50)	<0.01	97.8	0.0	2.2
	Paid intercourse	1.07 (0.27)	2.76 (0.57)	<0.01	94.2	1.4	4.3
	Non-commercial intercourse	2.33 (0.84)	1.53 (0.74)	<0.01	81.2	2.9	15.9
Behaviors by number of partners	Seduction	2.87 (0.35)	1.08 (0.29)	<0.01	92.8	3.6	3.6
	Non-genital foreplay alone	2.41 (0.72)	1.15 (0.43)	<0.01	91.3	1.4	7.2
	Non-genital foreplay in a couple	2.78 (0.48)	1.11 (0.41)	<0.01	93.5	0.0	6.5
	Non-genital foreplay in a group	2.04 (0.82)	1.59 (0.72)	<0.01	79.7	4.3	15.9
	Genital masturbation alone	2.72 (0.50)	1.12 (0.37)	<0.01	90.6	1.4	8.0
	Couple coitus	2.94 (0.29)	1.04 (0.26)	<0.01	97.8	0.0	2.2
	Group coitus	2.12 (0.82)	1.59 (0.73)	<0.01	77.5	6.5	15.9
	Anal masturbation alone	1.34 (0.59)	2.21 (0.81)	<0.01	87.0	2.2	10.9
	Penetrating anal in a couple	1.62 (0.78)	2.08 (0.90)	0.01	87.0	0.7	12.3
	Receiving anal in a couple	1.66 (0.77)	2.07 (0.87)	0.02	82.6	1.4	15.9
	Penetrating anal in a group	1.34 (0.63)	2.31 (0.87)	<0.01	94.2	0.0	5.8
	Receiving anal in a group	1.29 (0.61)	2.46 (0.76)	<0.01	90.6	0.0	9.4
	Oral sex alone	1.64 (0.81)	1.93 (0.88)	0.02	87.7	0.7	11.6
	Giving oral sex to a couple	2.74 (0.54)	1.10 (0.34)	<0.01	93.5	0.0	6.5
	Receiving oral sex from a couple	2.80 (0.48)	1.14 (0.42)	<0.01	90.6	2.2	7.2
	Giving oral sex in a group	2.13 (0.87)	1.63 (0.79)	<0.01	82.6	3.6	13.8
	Receiving oral sex in a group	2.28 (0.83)	1.50 (0.75)	<0.01	84.8	5.1	10.1
Associated elements	Pornography use alone	2.16 (0.76)	1.45 (0.65)	<0.01	76.8	1.4	21.7
	Pornography use in a couple	2.06 (0.77)	1.46 (0.68)	<0.01	79.7	2.9	17.4
	Pornography use in a group	1.62 (0.77)	1.84 (0.85)	0.08	88.4	0.7	10.9
	Use of erotic toys alone	2.37 (0.79)	1.27 (0.54)	<0.01	89.1	2.2	8.7
	Use of erotic toys in a couple	2.53 (0.66)	1.21 (0.49)	<0.01	87.7	1.4	10.9
	Use of erotic toys in a group	1.90 (0.83)	1.59 (0.79)	0.01	87.0	1.4	11.6
	Drug use in sex alone	1.28 (0.53)	2.17 (0.89)	<0.01	93.5	0.0	6.5
	Drug use in sex in a couple	1.56 (0.75)	1.79 (0.83)	0.56	78.3	3.6	18.1
	Drug use in sex in a group	1.56 (0.75)	2.07 (0.88)	<0.01	84.8	4.3	10.9
Paraphilia	Exhibitionism	1.12 (0.39)	2.72 (0.61)	<0.01	93.5	1.4	5.1
	Fetishism	1.45 (0.68)	1.98 (0.78)	<0.01	84.8	1.4	13.8
	Frotteurism	1.06 (0.29)	2.87 (0.46)	<0.01	96.4	1.4	2.2
	Relative sex intercourse	1.09 (0.37)	2.81 (0.53)	<0.01	96.4	1.4	2.2
	Masochism	1.34 (0.62)	2.49 (0.74)	<0.01	87.0	2.2	10.9
	Sadism	1.15 (0.45)	2.68 (0.64)	<0.01	94.2	1.4	4.3
	Transvestism	1.12 (0.38)	2.31 (0.87)	<0.01	97.1	0.0	2.9
	Voayeurism	1.46 (0.68)	2.33 (0.80)	<0.01	80.4	5.8	13.8

*No dissonance: one or two of the two options were answered at the lowest level; low dissonance: one of the options was scored higher, but the other was also marked at the lowest level; high dissonance: avoiding and approaching were answered with the same intensity.

The average scores showed a sample variation of responses either in approaching (Some: *M* = 10.84, SD = 4.54; High: *M* = 18.28, SD = 6.32; *t* = −9.86, *p* < 0.01) or avoiding values (Some: *M* = 9.24, SD = 5.21; High: *M* = 15.85, SD = 6.45; *t* = −9.56, *p* < 0.01) (Table 3), with a higher ED being the most common in both factors.

**Table 3:** Subject’s average count of the 51 behaviors for each intensity of approach and avoidance.

ED	None	Some	High	*p*-value
Approaching ED	*M* = 21.76; SD = 6.55	*M* = 10.84, SD = 4.54	*M* = 18.28, SD = 6.32	Some vs. High: *t*-test = −9.86, *p* < 0.01
Avoiding ED	*M* = 25.75; SD = 8.45	*M* = 9.24, SD = 5.21	*M* = 15.85, SD = 6.45	Some vs. High: *t*-test = −9.56, *p* < 0.01

M, Mean; SD, standard deviation.

This difference was also seen in the reporting of more behavior with high dissonance (*M* = 5.51, SD = 4.47) than low dissonance (*M* = 1.05, SD = 1.69) (*t* = −11.28, *p* < 0.01) (Table 4). Nevertheless, the absence of dissonance was the most common outcome within the participants’ repertoire (*M* = 44.30, SD = 4.49) (Table 4).

**Table 4:** Subject’s average count of the 51 behaviors for each dissonance level.

ED	None	Low	High	*p*-value
Dissonance	*M* = 44.30, SD = 4.49	*M* = 1.05, SD = 1.69	*M* = 5.51, SD = 4.47	Low vs. High: *t*-test = −11.28, *p* < 0.01

M, Mean; SD, standard deviation.

In addition, no significant results were found in the univariate association analysis with the general description variables (Table 5), except for the variable “traumatic event experienced.”

**Table 5:** Associations between dissonance levels and overall sexual life description variables.

Sexual features	No dissonance	Low dissonance	High dissonance
General satisfaction (1–5) (Mean = 3.40; SD = 1.01)	Rho: −0.48 *p* > 0.05 *n* = 134	Rho: −0.12 *p* > 0.05 *n* = 134	Rho: 0.06 *p* > 0.05 *n* = 134
Self-attractiveness perceived (1–5) (Mean = 3.99; SD = 0.80)	Rho: 0.48 *p* > 0.05 *n* = 138	Rho: −0.11 *p* > 0.05 *n* = 138	Rho: 0.02 *p* > 0.05 *n* = 138
Self-mastery perceived (1–5) (Mean = 3.69; SD = 0.91)	Rho: 0.47 *p* > 0.05 *n* = 138	Rho: −0.02 *p* > 0.05 *n* = 138	Rho: −0.01 *p* > 0.05 *n* = 138
Traumatic experience Yes, *n* = 42 (30.4%) No, *n* = 93 (67.4%) No response, *n* = 3 (2.2%)	Chi-Square: 92.06 *p* < 0.01 *n* = 138	Chi-Square: 25.57 *p* < 0.05 *n* = 138	Chi-Square: 75.6 *p* < 0.01 *n* = 138
Number of intercourses per month (last 12 months) (Mean = 3.33; SD = 5.82)	Pearson: −0.00 *p* > 0.05 *n* = 136	Pearson: 0.01 *p* > 0.05 *n* = 136	Pearson: 0.03 *p* > 0.05 *n* = 136
Number of partners in the last 12 months (Mean = 6.88; SD = 9.17)	Pearson: 0.11 *p* > 0.05 *n* = 138	Pearson: −0.14 *p* > 0.05 *n* = 138	Pearson: −0.08 *p* > 0.05 *n* = 138

**Figure 1:**
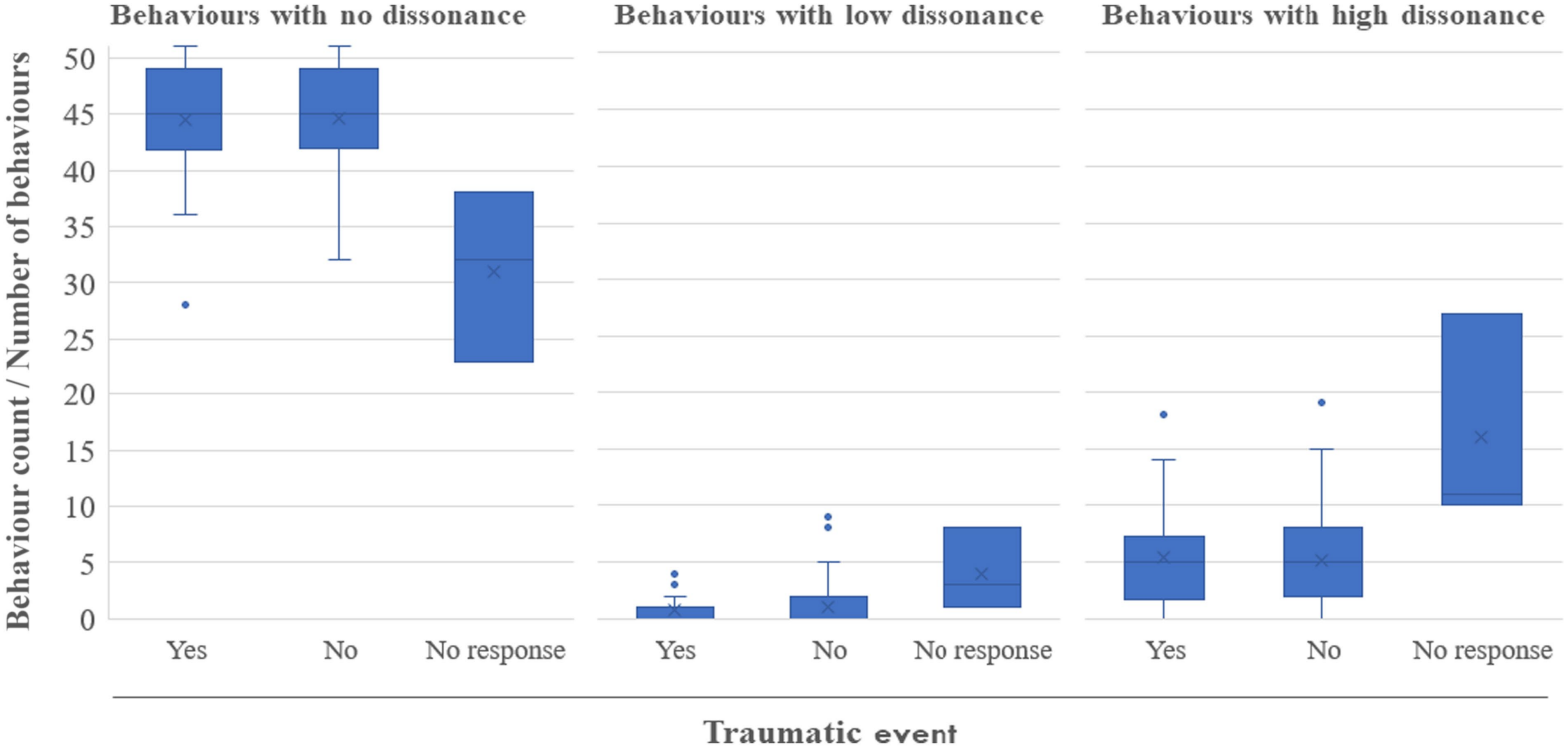
Associations between traumatic event responses and dissonance level counts. Number of subjects: Yes = 42 (30.4%); No = 93 (67.4%); No response = 3 (2.2%).

In this case, only three participants chose the “no response” option. Figure 1 shows how the responses presented to those who were not willing to answer that particular question reflected a higher score on the two dissonance options. On the other hand, we found almost the same scores among the groups who were willing to respond with either “yes” or “no,” with no significant differences when removing the three “no response” subjects (*p* > 0.05).

Variable selection revealed differences among the three options assessed (Table 6). The approach ED yielded 17 behaviors, accounting for 99.2% of the variance observed, while the reject or avoid ED resulted in 29 behaviors and 99.5%, not necessarily matching the approaching variable. The same difference was observed in dissonances, where an “unlikely” set of 17 behaviors produced 99.7% of the variability.

**Table 6:** Results of the unsupervised variable selection analysis loading for 99% of the variability of the approaching, avoiding, and dissonance sets independently.

		% of variance explained		
		APPROACH	AVOID	DISSONANCE
Partner type taxis	Biological man taxis	2.34		
	Biological woman taxis	12.61	2.94	2.66
	Masculine attitude taxis	2.37		
	Feminine attitude	7.67	2.71	
	Athletic body type			2.83
	Lean body type			
	Plump body type			
	Casual partner	1.89	0.74	
	Trusted partner without emotional bond	1.28	0.43	5.04
	Steady relationship partner			
	Child age			
	Adolescent age			
	Young adult age			
	Mature age			
	Elder age			
	Paid intercourse		1.41	
	Non-commercial intercourse			
Behaviors by number of partners	Seduction			
	Non-genital foreplay alone			
	Non-genital foreplay in a couple			1.88
	Non-genital foreplay in a group	9.85	3.73	7.23
	Genital masturbation alone			
	Couple coitus			
	Group coitus	13.38	5.80	5.02
	Anal masturbation alone		5.77	
	Penetrating anal in a couple	2.69	4.28	
	Receiving anal in a couple		4.31	
	Penetrating anal in a group	4.99	7.79	
	Receiving anal in a group	1.49	6.43	
	Oral sex alone		2.13	3.17
	Giving oral sex to couple			0.53
	Receiving oral sex from couple			0.58
	Giving oral sex in a group	13.21	7.95	7.43
	Receiving oral sex in a group	8.63	6.01	7.71
Associated elements	Pornography use alone		2.02	4.14
	Pornography use in a couple		2.07	12.73
	Pornography use in a group	3.59	5.13	6.55
	Use of erotic toys alone		0.62	6.17
	Use of erotic toys in a couple		1.75	13.29
	Use of erotic toys in a group	8.23	5.72	12.77
	Drug use in sex alone		2.79	
	Drug use in sex in a couple		2.11	
	Drug use in sex in a group	3.43	6.89	
Paraphilia	Exhibitionism		0.29	
	Fetishism		3.22	
	Frotteurism			
	Relative sex intercourse			
	Masochism		1.55	
	Sadism			
	Transvestism		1.06	
	Voayeurism	1.55	1.87	
**Total %**		**99.20%**	**99.52**	**99.73**
**Total vv**	**51**	**17**	**29**	**17**

Finally, we conducted a cluster analysis for the approach and avoidance ED separately. The variable selection analysis included all the items evaluated in the subjects. From the total set, without anticipating any latent variables or theoretical groupings, this selection indicated which stimuli accounted for more than 99% of the variance in the participants’ responses, thereby identifying the most relevant patterns for finding differences between them. Therefore, these variables, identified as significant for differentiation between individuals, were explored in terms of grouping both by item and subject.

**Figure 2:**
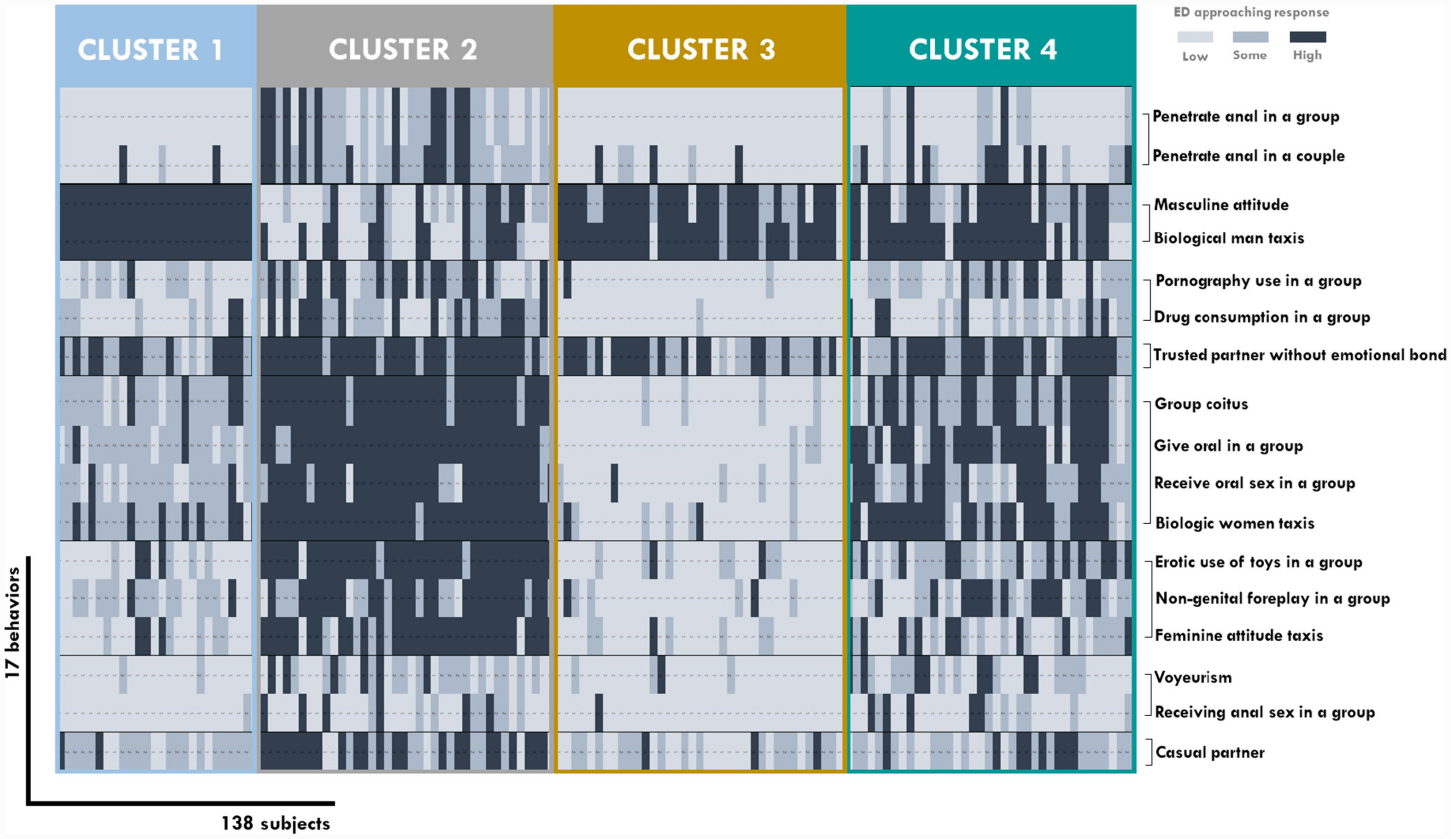
Outcomes of approaching ED cluster analysis using variables resulted in unsupervised variable selection.

A total of four clusters (groups of subjects) and eight classes (groups of behaviors) were selected with the highest ICL-BIC = −2,400 for the approaching ED: 1: anal in a group and anal in a couple; 2: masculine attitude and biological man trait; 3: pornography use and drug consumption in a group during sex; 4: trusted partner without emotional bond; 5: group coitus, giving and receiving oral sex within a group, and biological woman trait; 6: non-genital foreplay and the use of toys in a group, plus feminine attitude trait; 7: voyeurism and receiving anal sex in a group; 8: casual partner (Figure 2). The model converged according to the gamma and pi estimates.

**Figure 3:**
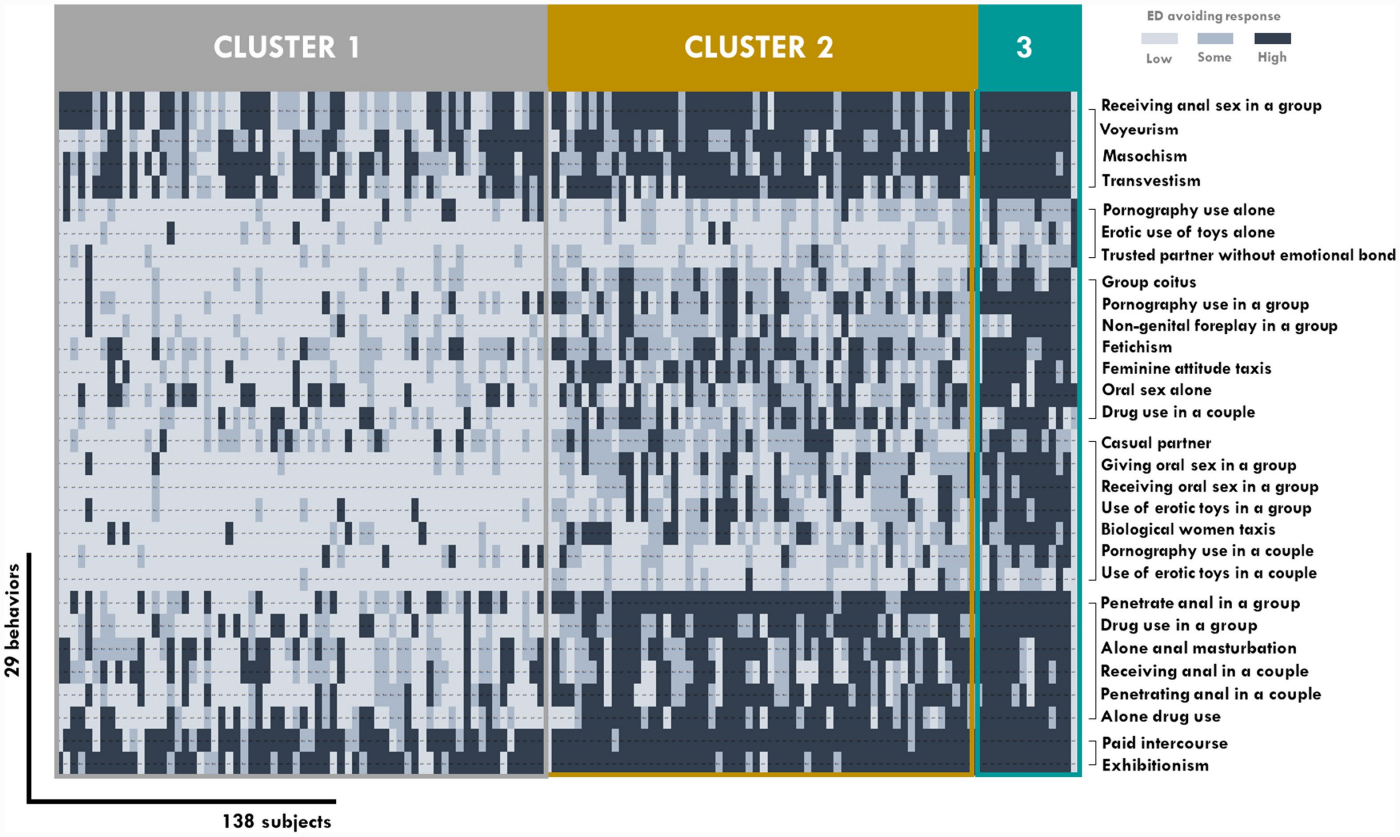
Outcomes of avoiding ED cluster analysis using variables resulted in unsupervised variable selection.

Finally, for avoidance of ED, a total of three clusters and six classes were selected with the highest ICL-BIC = −3,200: 1: voyeurism, masochism, transvestism, and receiving anal sex in a group; 2: pornography use alone, use of toys, and intercourse with a trusted partner without emotional bond; 3: group coitus, use of pornography, non-genital foreplay in a group, fetishism, feminine attitude trait, oral sex alone, and drug use in a couple; 4: a casual partner, giving and receiving oral sex in a group, the use of toys in a group, the biological woman trait, and use of pornography and toys in a couple; 5: anal penetration and drug use in a group, alone anal masturbation and alone drug use, and receiving and penetrating anal sex in a couple; 6: paid intercourse and exhibitionism (Figure 3). The model converged according to gamma and pi estimates.

## Discussion

Our study aims to explore ED using the ordinal scale, which is easily categorized, i.e., “low,” “some,” and “high,” each specifying a set of behaviors to compare and give personal anchored responses.

This design follows Yannakakis et al.’s (2021) explanation of how the measures of emotional intensity are variables that usually show a poor expression of graded experienced values when isolated but a good categorization based on an internal context and given an anchor. To our knowledge, the use of an anchored, exhaustive, and commonly shared repertoire (Velo et al., 2023) is the first attempt to screen for ED in detail using a comprehensive set of sexual actions and contextual elements.

The present study aims to provide further evidence for a better understanding of an individual’s ED, which is likely to be associated with sexual appraisal (Madan, 2017), modulation of response (Driscoll et al., 2018; Ferrey et al., 2012), and eventual behavior (Driscoll et al., 2018; Ferrey et al., 2012).

In general terms, our results show how the participants consistently tended to appraise and express higher values for sexual behavior, either positively or negatively. In the sample presented, response patterns consistently showed that, given the same graduation and anchoring of options, participants tended to respond by feeling attracted or repelled in the most intense way. Additionally, we found that dissonance is not a major but a common trend in the sample. In fact, this dissonance was usually shown in some way in the vast majority of subjects we surveyed, and when it did appear, it was more likely to do so at a “high” level of dissonance. Possibly, the relevance, social significance, and intimacy required for sexual behavior enable the tendency to feel a greater impulse to approach or withdraw. In any case, the present study does not show the acquisition or vital variation of these EDs but simply provides a snapshot of a measurement in different subjects who seem to coincide in terms of the general pattern.

Looking into this dissonance, we explored its relationship with general descriptors of sexual experience but did not find any univariate associations between them. Therefore, in light of these results, we did not find any support for the idea that this dissonance was an unusual or surprising emotional process. We discuss our results in such a way that it is a natural experience in life, common, and apparently unrelated to any general satisfaction or self-perception. However, further studies are required to verify the accuracy of our discussion using different samples, life development stages, and other cultural and personal factors.

Having established an overall picture in general terms, the next part of the exploration consisted of identifying the minimum number of behaviors that explained the main differences between participants. Cabanac (2002) discussed the “common currency” to apply intensity and hedonic value to reality, and we wanted to explore this further and look for the exchange values of traded currencies.

This objective was assessed through unsupervised variable selection for each ED, i.e., approaching/avoiding/dissonance, and it was concluded that the participants differed mostly in a specific set of stimuli, which also varied when considering a positive or negative ED (Table 6).

Thus, these results led to two sets of behaviors in each factor (Approach and Avoid): one set where the variations did not load for differences (Table 6) and could be identified as a commonly shared trend, and another where the individual’s preference emerged and distinguished the subjects more accurately than the prior one. This finding could be taken as a sign of different classes of behavior, where it is possible to find a set common to the majority of people and other classes of less common conduct, which are able to point out the main differences between the subjects.

The importance of this result lies in the fact that it does not seem to be sufficient to evaluate a subject in relation to a behavior or in relation to a behavior that attracts them. A person’s hedonic profile can vary depending on whether they are approaching or avoiding something. This result makes the analysis of sexual behavior much more complex, as it could mean that the variables that people combine in their perception of their sexual activity are multiplied, comparing the intensity of positive sensations with the intensity of negative ones before making behavioral decisions.

The same applies to dissonance. In the present study, we relegated the importance of this factor to the background, as we found no relationship with the general markers we evaluated. However, for future research, it is important to note that in our sample, the behaviors that best explain the differences in dissonance do not necessarily coincide with those that explain attraction or rejection.

Finally, following the selection results, we conducted a more complex analysis to explore the ensemble data. With this, we sought to complement the results of behaviors with a preliminary exploration of hedonic profiles. For this purpose, cluster analysis was used to screen group patterns using sets of behaviors that accounted for 99% of the variance. In this final step, we also found significant results: four clusters for approaching the ED and three clusters for avoiding the ED.

In the first cluster, regarding the approaching subsets (Figure 2), the eight classes suggested patterns of desire that could accurately shape the hedonic key to differentiate an individual’s profiles. Thus, a member of cluster three could be identified as a man with a manly attitude, interested in emotional connection, and not attracted to group actions, drug or porn consumption in the company, or unusual partners. Similarly, a subject allocated to cluster three could be identified as someone who enjoys group intercourse and feminine women and is less interested in anal sex and voyeurism.

The importance of this result lies in pointing to behaviors such as anal sex with a partner or in a group, associated use of drugs and/or toys, the masculinity or femininity of the partner, or the temporary nature of that partnership as the main elements that differentiate the participants.

It is expected that the clustering pattern would show results if all variables were included; however, these results would not necessarily be useful in differentiating subjects. We believe that variable selection analysis enables clustering using the most relevant variables. Thus, the results presented can be read not as global profiles but as indicators of relevant hedonic patterns.

It is also relevant to comment that we have decided not to name these groups using a word commonly used in everyday language in order to avoid misunderstandings based on everyday criteria, such as moral judgments, social clichés, or any other labels that could be used at such an early stage of validation of this profiling proposal.

Similarly, a profile differentiation was provided with rejection behavior, although we considered it important to note the long list of possible actions included and subsets found. It appears that the clusters found differentiate profiles by a tendency toward generalized rejection. In this case, we observed fewer specific combinations and more general positioning toward a larger set of elements.

This specific feature leads to distinctions mainly among the large rejecters, intermediates, and non-rejecter clusters. We found stronger patterns in terms of anal actions, intercourse that had been paid for, and some paraphilias such as exhibitionism, masochism, transvestism, and voyeurism. Despite this, the results of our sample indicate a more consistent pattern in terms of rejection than attraction profiles.

## Conclusion

In this study, we report four pieces of evidence for ED in the context of sexual behavior which lead us, first, to consider both positive and negative motivations graded and anchored in a delimited repertoire, instead of univariate and/or qualitative expressions without a framework; second, to take into account the usual experience of dissonance drive in sexual behavior prior to concluding the positive or negative experiences; third, to the use of anchored and wide information to define population trends in sexual behavior, indicating the specific sets where some account for the variance and are more relevant to explain the differences; and, finally, the likely cluster division of subjects in terms of ED when comparing behavior, in order to find accurate profiles and to be able to compare with suitable individuals.

Our findings do not necessarily mean that our patterns will be expressed in exactly the same distribution when using a larger sample or a culturally different one, but we present them as early evidence of the need to assess the whole picture of ED loads, including dissonance, and to take into account the importance of assessing them within an anchor frame to improve the conclusions regarding influence, causation, or relevance for many sexual behavior outcomes.

We believe that this is a deep classification of ED, which could draw distinctions between profiles and play an important role in the description of behavioral patterns, for which we conclude the importance of further research.

### Limitations

The first limitation of this study is the exploratory condition, which affects some of the plotted methods used. As an exploratory result, we did not find any similar previous results with which we could compare the analysis or any sample description carried out on this specific topic; therefore, we cannot assure the best adequacy of our process. Furthermore, our sample was not large or diverse enough to consider the results as accurate ranges within the general population but only to screen trends that must be studied in depth in future studies.

Finally, we did not compare a list of behaviors set in a randomly ordered disposition between the subjects; thus, we could not identify the tendencies of responses related to the given anchor. We believe that this issue should be considered in future research beyond the exploratory objectives of this study.

## Data Availability

The raw data supporting the conclusions of this article will be made available by the authors, without undue reservation.
